# Application of portable XRF and VNIR sensors for rapid assessment of soil heavy metal pollution

**DOI:** 10.1371/journal.pone.0172438

**Published:** 2017-02-24

**Authors:** Bifeng Hu, Songchao Chen, Jie Hu, Fang Xia, Junfeng Xu, Yan Li, Zhou Shi

**Affiliations:** 1 Institute of Agricultural Remote Sensing and Information Technology Application, Zhejiang University, Hangzhou, China; 2 INRA, InfoSol Unit, Ardon, Orléans, France; 3 UMR SAS, INRA, AGROCAMPUS OUEST, Rennes, France; 4 College of Science, Hangzhou Normal University, Hangzhou, China; 5 Institute of Land Science and Property Management, School of Public Affairs, Zhejiang University, Hangzhou, China; RMIT University, AUSTRALIA

## Abstract

Rapid heavy metal soil surveys at large scale with high sampling density could not be conducted with traditional laboratory physical and chemical analyses because of the high cost, low efficiency and heavy workload involved. This study explored a rapid approach to assess heavy metals contamination in 301 farmland soils from Fuyang in Zhejiang Province, in the southern Yangtze River Delta, China, using portable proximal soil sensors. Portable X-ray fluorescence spectroscopy (PXRF) was used to determine soil heavy metals total concentrations while soil pH was predicted by portable visible-near infrared spectroscopy (PVNIR). Zn, Cu and Pb were successfully predicted by PXRF (R^2^ >0.90 and RPD >2.50) while As and Ni were predicted with less accuracy (R^2^ <0.75 and RPD <1.40). The pH values were well predicted by PVNIR. Classification of heavy metals contamination grades in farmland soils was conducted based on previous results; the Kappa coefficient was 0.87, which showed that the combination of PXRF and PVNIR was an effective and rapid method to determine the degree of pollution with soil heavy metals. This study provides a new approach to assess soil heavy metals pollution; this method will facilitate large-scale surveys of soil heavy metal pollution.

## Introduction

Soil contamination with heavy metals has become a worldwide environmental issue [1–4] and there are serious problems with soil heavy metals pollution in China. According to The National Soil Pollution Condition Investigation Communique released by the Ministry of Environmental Protection and Ministry of Land and Resources on April 17th, 2014, the proportion of contaminated samples in China is 16.1% [5]. There is an urgent need to complete high-density soil sampling to determine the boundaries of contaminated areas and then to prevent and control further soil pollution. Traditional laboratory analyses of heavy metals in soils, such as AFS (Atomic Fluorescence Spectrometry), AAS (Atomic Absorption Spectrometry) and ICP-OES (Inductively Coupled Plasma Optical Emission Spectroscopy), are time-consuming, laborious and expensive, making them unsuitable for large-scale, rapid and high-density assessment of soil heavy metals contamination. Portable X-ray fluorescence (PXRF) has been considered as an effective technique to measure total concentrations of soil heavy metals based on linear regression models between fluorescence intensity and concentration of specific heavy metals[6]. In addition, it can rapidly and nondestructively quantify various heavy metals at the same time. Therefore, PXRF has been widely applied by many researchers in several fields [7–10].

Availability of heavy metals in soils determines their bioavailability and environmental toxicity; measurement of available heavy metals is a better indicator of metals content than total heavy metals. Soil pH is a critical factor controlling the activity of heavy metals in soils [11], therefore it is very important to measure heavy metal contents and the corresponding pH values of samples at the same time when evaluating soil heavy metals contamination. The feasibility of PXRF for rapid measurement of soil heavy metals has been reported [12–16].

VNIR has been used to predict soil properties, including pH, soil carbon and soil nitrogen [17–18]. However, few studies have rapidly evaluated soil pH and heavy metals concentrations at the same time let alone rapidly assessed composite heavy metal pollution grades. Thus, this paper investigated the feasibility of rapid assessment of heavy metals contamination in soils by PXRF and PVNIR sensors as a means to provide a rapid, easy, non-destructive means of evaluating soil heavy metals contamination at large scale. This has great significance for improving soil environmental quality and ensuring food security.

## Materials and methods

### 2.1 Study region

**Fig 1:**
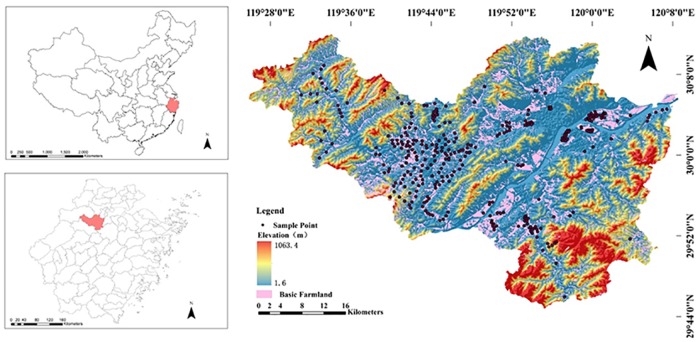
Location of the study area.

Fuyang district is located in the southern part of the Yangtze River Delta, in Zhejiang Province, China (shown in Fig 1), with a land area of about 1831 km^2^ (29°44'4"–30°11'58.5"N and 119°25'00"–120°19'30"E), of which hilly terrain accounts for 75.9%, plains account for 17%, and water bodies account for 5.4%. It belongs to the subtropical monsoon climate influenced region with an annual average temperature of 17.80°C and an average annual rainfall of 1486.80 mm. There are 24,000 ha of basic farmland, 800 ha of grain production zones and 6,000 ha of modern agriculture parks currently in Fuyang, making it one of the most important agricultural grain production regions in the Yangtze River Delta. With this rapid economic growth, especially industrial growth, arable land in Fuyang district faces great risk of soil heavy metals pollution [19–20]. Permissions to conduct research in Fuyang district were obtained from the owners of the land. The field studies not involve endangered or protected species. And since it is a regularly survey no specific permissions were required for these locations.

### 2.2 Soil sampling and laboratory analysis

A total of 301 soil sites were selected on arable land in Fuyang, Zhejiang Province, China in 2011. At each site, five individual core samples were taken from the topsoil (0–20 cm) layer, using a random sampling design within a 10 × 10 m area. These core samples were combined to obtain a composite soil sample for each site. Soil samples were air-dried and ground to pass a sieve with a mesh of 100 before laboratory chemical analysis and spectral measurements.

Analysis were conducted with reference to the Technical Specification for Soil Environmental Monitoring (TSSEM) [21] published by the State Environmental Protection Administration of China and Environmental Quality Standard for Soils (EQSS) [22]. Soil pH and eight heavy metals including chromium (Cr), lead (Pb), cadmium (Cd), mercury (Hg), arsenic (As), copper (Cu), zinc (Zn) and nickel (Ni) were analyzed. Soil pH was determined by pH meter in water:soil at a ratio of 2.5:1. Concentrations of Pb, Cu, Zn and Ni were determined by atomic absorption spectrometry after dissolution with hydrochloric acid, hydrofluoric acid, nitric acid and perchloric acid. As content was measured using atomic fluorescence spectrometry after soil digestion with nitric acid hydrochloride.

### 2.3 Heavy metal content measurements of PXRF

**Fig 2:**
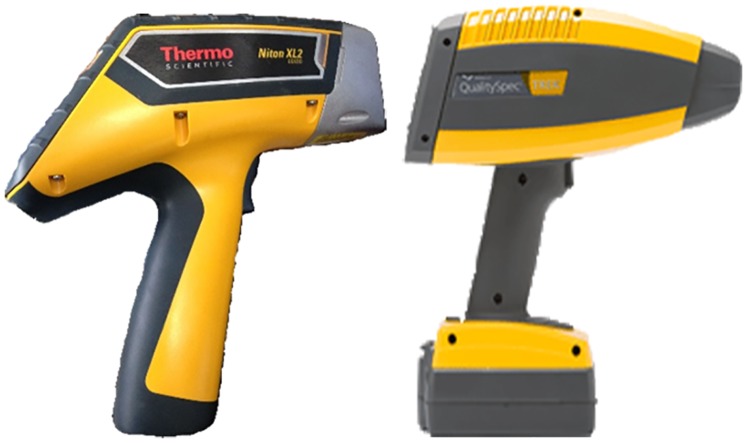
PXRF and portable VNIR analyzers.

Depending on the calibration model between spectral information and the component to be measured, XRF is a technique used for the prediction of components indirectly in unknown samples [23]. PXRF has large quantity of advantages including its portability, ease of use, low cost, high speed, high accuracy and non-destructive detection; it can detect multiple elements at the same time. A Niton XL2 GOLDD XRF Analyzer (Thermo Fisher Scientific Inc., Waltham, MA, USA) was used in this study (Fig 2). The instrument was equipped with an Ag anode operating at a maximum of 45 kV and 80 μA. All soil PXRF measurements were performed with a portable test stand in the laboratory. The total concentrations of soil heavy metals were calculated through the built-in algorithm under Soil Mode in the PXRF. Before measurements, ground soils were placed in a 31-mm X-ray sample cup and were then covered with an X-ray film. Three parallel samples were measured for each soil sample and the average results were considered as the concentrations of heavy metals in soil samples. For all the measurements, the measurement time was 90 s.

Detection are sensitive to the specified elements, so the limits of detection (LOD) for each soil heavy metal varies using PXRF. For Cd and Hg, LODs were much higher than the guideline threshold concentrations in arable land defined in the EQSS, and the LOD of Cr was also quite close to the guideline threshold concentration, so in this study we focused on the other five heavy metals, including Pb, As, Cu, Zn and Ni [24].

**Fig 3:**
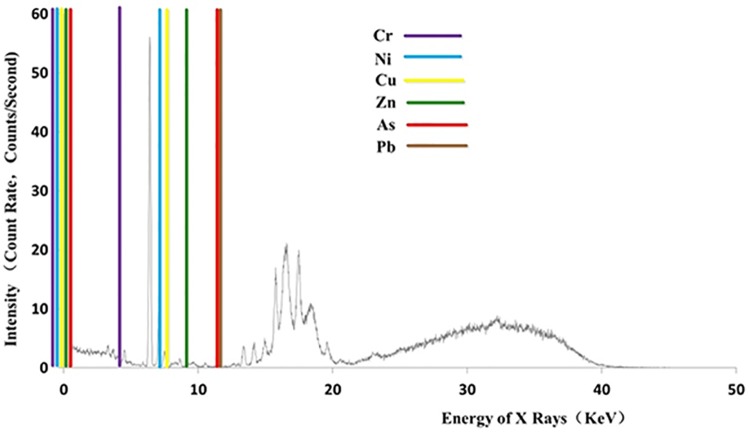
Representative soil spectrum of XRF, highlighting the qualification ranges of soil heavy metals.

A representative soil spectrum obtained from PXRF was shown in Fig 3; ‘counts’ represent the emitted spectrum intensity at each photon energy, so they are the basis for quantitative analysis as well as the built-in algorithm. Characteristic X-ray fluorescence spectrum energies of Cr, Ni, Cu, Zn, As, Pb are 0.583, 0.869, 0.93, 1.035, 1.035 and 12.614 Kev, respectively, at Lb1 and 5.947, 8.265, 8.905, 9.572, 11.726 and 84.936 Kev, respectively, at Kb1.

### 2.4 pH measurements of VNIR spectra

Under laboratory conditions, soil VNIR spectra were collected by a QualitySpec Trek Portable Spectrometer (ASD Inc., Boulder, CO, USA), which has a spectral range of 350 to 2500 nm and a resolution of 3 nm at 700 nm, 9.8 nm at 1400 nm and 8.1 nm at 2100 nm. Before measurement, the instrument was corrected with a white reference and soil samples were placed in a Petri dish 10 cm in diameter and 1.5 cm deep. For each soil sample, 10 spectra were collected and then they were averaged in to one spectrum to represent the spectra of that soil sample.

### 2.5 Division of calibration and validation datasets

In this study, soil samples were separated into two divisions: two-thirds of which were calibration dataset (201 soil samples) and the remaining third for individual validation (100 soil samples). The calibration dataset was used to build the prediction model while the validation dataset was applied to evaluate the ability of the prediction model. Soil samples in calibration and validation datasets were determined by the Rank-Kennard-Stone (Rank-KS) algorithm [25]. The detail of this algorithm was as follows: first, soil samples were ranked in ascending order of pH concentrations; second, the soil samples were divided into *n* average partitions (the last partition might be smaller than other partitions); after that, the Kennard-Stone algorithm was performed in each partition to generate subsets of calibration and validation datasets; finally, all subsets were merged into calibration and validation datasets. This algorithm selected representative soil samples with enough variation in spectra and pH concentrations to build a robust prediction model and boost predictive ability. The number of partitions was optimized to 10 in this study.

### 2.6 Partial least squares regression

Partial least squares regression (PLSR), a classical non-parametric linear regression method, has been widely used in spectral analysis. The method searches a linear regression model in a new dimension that was projected by the predictors and a dependent variable. When reducing the numbers of predictors, PLSR also decreases the risk induced by multicollinearity of predictors. Therefore, PLSR is a good choice for analyzing soil spectra that contain thousands of wavelengths. To avoid over-fitting or under-fitting, leave-one-out cross validation (LOOCV) was applied to optimize the number of latent variables in the calibration [26–28].

### 2.7 Accuracy assessment and variable selection

For the regression model, the coefficient of determination (R^2^), the root-mean-square error of prediction (RMSE) and the residual estimation deviation (RPD) are used to assess the accuracy of the prediction model. A model with high R^2^ and RPD and low RMSE is considered a good model. RPD has been defined to three classes [29]: category A (RPD >2) models can provide accurate predictions, category B (1.4≤ RPD ≤2) models can provide moderately accurate predictions and category C (RPD <1.4) models have no prediction ability.

For classification, we assessed the performance of the models using prediction accuracy and Cohen's Kappa coefficient (*k*). Good classification is always accompanied with a high prediction accuracy and Kappa coefficient. Generally, Kappa coefficient has been defined to five classes [30]: category A (*k* >0.8) means almost perfect agreement; category B (0.8≥ *k* >0.6) means substantial agreement; category C (0.6≥ *k* >0.4) means moderate agreement; category D (0.4≥ *k* >0.2) means fair agreement; and category E (*k ≤*0.2) means slight agreement.

Variable importance in the projection (VIP) was applied to select variables. The band with a high VIP score has a substantial contribution to the model.

### 2.8 Method of soil heavy metal pollution assessment

Considering the effect of each heavy metal on soils and highlighting the influence of high concentrations of heavy metals on soil quality, we referenced TSSEM and EQSS and established procedures for assessing soil heavy metal as follows: first, soil pH values were defined to three classes: <6.5, 6.5≤ pH ≤7.5 and >7.5; second, the pollution threshold for each soil heavy metal was determined by land use (paddy fields here) and pH class [22]; third, the single pollution index (*SPI*) for each heavy metal was determined (Eq 1); finally, the Nemerow composite pollution index (*NCPI*) was calculated (Eq 2).

$$P_{i}=\frac{C_{i}}{S_{i}}$$ (1)

where *C*_*i*_ was the concentration of soil heavy metal *i* and *S*_i_ was the pollution threshold of *i*.

$$NCPI=\sqrt{\frac{{\left(P_{imax}\right)}^{2}+{\left({\bar{P}}_{i}\right)}^{2}}{2}}$$ (2)

where *P*_*max*_ was the maximum value among *NPI* of each heavy metal and $\bar{P}$ was the mean *NPI* of each heavy metal.

As *NCPI* was a comprehensive index, it was used to classify the soils in terms of heavy metal pollution. Table 1 shows the classifications of soil heavy metal pollution.

**Table 1:** Classes of soil heavy metal pollution[31–33].

Class	*NCPI*	Grade	Description of soil heavy metal pollution
1	≤0.7	Safety	Clean
2	0.7< *NCPI* ≤1.0	Alert	Slight clean
3	1< *NCPI* ≤2.0	Slight pollution	Soil pollution exceeds background, crops start to be polluted
4	2< *NCPI* ≤3.0	Moderate pollution	Soils and crops have been polluted moderately
5	*NCPI* >3.0	Severe pollution	Soils and crops have been polluted severely

## Results and discussion

### 3.1 Descriptive statistics of soil heavy metals

The summary statistics of the total concentrations of the elements in the soils are shown in Table 2. The observed range in the concentrations of Zn, Ni, Cu, Pb and As in the soil were 59–4194, 2–114, 8–297, 15–159 and 1–53 mg/kg, respectively. The mean concentrations of Zn, Ni, Cu, Pb and As were 162.26, 17.96, 35.80, 30.31, 6.61 and 8.39 mg/kg, respectively.

**Table 2:** Descriptive statistics for heavy metal concentrations in soils (mg/kg).

Statistics	pH	Zn	Ni	Cu	Pb	As
Mean	5.85	162.26	17.96	35.80	30.31	8.39
SD	1.19	285.90	12.07	33.36	20.10	5.27
Min	3.55	59.00	2.00	8.00	15.00	1.00
Max	8.00	4194.00	114.00	297.00	159.00	53.00
CV(%)	20.31	176.20	67.22	93.18	66.31	62.83
SBC_1_^a^		70.6	24.6	17.6	23.7	9.2
SBC_2_^b^		74.2	26.9	22.6	26.0	11.2

^a^ SBC_1_, soil background content in Zhejiang Province;

^b^ SBC_2_, soil background content in China; SD, standard deviation.

The coefficient of variation (CV) indicates the degree of variability for the concentrations of metal in the soil. CV ≤20% is regarded as low variability, 21%< CV ≤50% is moderate variability, 50%< CV ≤100% is high variability, and CV above 100% is exceptionally intense variability. The CV of metals in urban soils in decreasing order were Zn (176.20%), Cu (93.18%), Ni (67.22%), Pb (66.31%) and As (62.83%). Zn showed exceptionally intense variability. This indicated that the concentrations and distribution of Zn were highly controlled by anthropogenic activities. Cu, Ni, Pb and As showed high variability, which suggested that anthropogenic activities were the main factor influencing the concentrations and distribution of Cu, Ni, Pb and As in the study area.

### 3.2 Soil pH prediction results using VNIR spectra

Five different spectral pretreatment methods (Savitzky-Golay denoising, SG; First-order difference, FD; Absorption rate, ABS; Multiple Scatter Correction, MSC; Standard Normal Variation, SNV) were used before PLSR modelling in this study. The prediction accuracies of different spectral pretreatment methods were shown in Table 3. The combination of SG and SNV obtained the best prediction result with an RMSE of 0.59 and an RPD up to 2.00. These results showed that the model with pretreatment by SG and SNV could qualitatively predict pH and almost reach the level of quantitative prediction, so this model was chosen as the optimized PLSR model for pH classification in this study. In the region with pH <6.5, the prediction result showed a relatively low degree of underestimation. Most of the sample points with measured pH values <6.5 had prediction pH values <6.5; for samples with pH values between 6.5 and 7.5 and pH values >7.5, the predicted pH values were generally underestimated. Most of the sample points with measured pH values of between 6.5 and 7.5 had predicted pH values <6.5 whereas most of the sample points with measured pH values >7.5 had predicted pH values between 6.5 and 7.5.

**Table 3:** Comparison between the prediction accuracy of PLSR models with different spectral preprocessing methods.

Items	SG+FD	SG	SG+ABS	SG+MSC	SG+SNV
RPD	1.59	1.70	1.77	1.84	2.00
RMSEP	0.72	0.67	0.65	0.62	0.59

RMSEP, The root mean square error of prediction.

**Fig 4:**
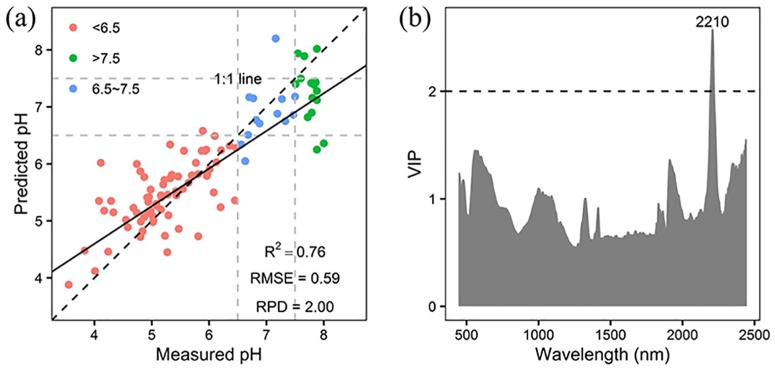
Prediction scatter plot of pH (a) and VIP scores of optimized PLSR model (b).

Fig 4(a) shows the scatter diagram of the optimal PLSR model, which demonstrates the trend of underestimation. The prediction VIP scores of different spectral bands of the optimized PLSR model were presented in Fig 4(b). Although it is one of the important properties of soil, pH does not have an associated specific soil spectral waveband; despite this, many studies have used this method and obtained good prediction results for soil pH. Chang et al. [29] thought that pH could be accurately predicted because it has a significant relationship with organic matter and clay content in soil. According to the VIP scores, the most important wavebands for the pH prediction model are the wavebands around 2210 nm. The wavebands near 2200 nm were significantly related to the frequency peaks of O-H and N-H. These wavebands were very important for predicting organic matter content so the prediction result for pH was similar to previous studies [34].

### 3.3 Soil heavy metals prediction results of PXRF

The detection limits of Ni, Cu, Zn, As and Pb in this study were 28, 22, 40, 9 and 12 mg/kg, respectively. The detection limit values of Zn, Pb and As were lower than the background values for Zhejiang Province and China, while the detection limit of Cu was higher than the background value of China but lower than the background value of Zhejiang Province. The detection limit value of Ni was higher than the background values of Zhejiang Province and China. All detection limit values were lower than the national corresponding standard value, so it is reasonable to use the PXRF method to measure concentrations of heavy metals and assess the grades of soil heavy metal pollution. The heavy metals concentrations of some soil samples were lower than the detection limit values, so we used the detection limit value to replace their concentration. This meant that the mean concentrations of soil heavy metals using PXRF were relatively higher than the chemical analysis results. The mean concentrations of Ni, Cu, Zn, As and Pb by PXRF were 39.13, 32.89, 132.40, 12.74 and 30.38 mg/kg, respectively (Table 4), all of which were higher than the background values of Zhejiang Province and China and also higher than the corresponding mean values of chemical analysis results (Table 4). This indicated that it is still necessary to lower the detection limit values of PXRF detectors to allow PXRF to be universally applied.

**Table 4:** Descriptive statistics for heavy metal concentrations in soils by PXRF methods (mg/kg).

	Zn	Ni	Cu	Pb	As
Mean	132.40	39.13	32.89	30.38	12.74
SD	134.39	13.84	23.50	32.23	4.67
Min	54.00	28.00	22.00	12.00	9.00
Max	1156.00	70.00	186.00	220.00	30.00
CV(%)	101.50	35.36	71.45	106.10	36.64
BV_1_^a^	70.6	24.6	17.6	23.7	9.2
BV_2_^b^	74.2	26.9	22.6	26.0	11.2
DLV^c^	40	28	22	12	9

Note:

^a^ BV_1_, Background value of Zhejiang Province;

^b^ BV_2_, Background value of China;

^c^ DLV, detection limit value of PXRF.

**Fig 5:**
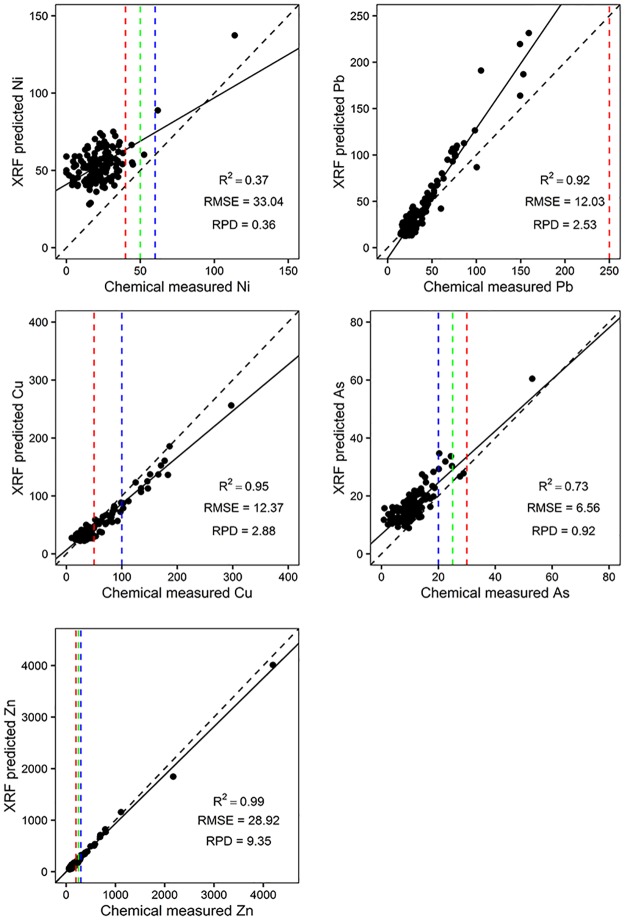
Regression of PXRF measurements against ICP-AES analysis for trimmed datasets. The red, green and blue dotted lines represent the national standard value of heavy metals when pH <6.5, 6.5–7.5 and >7.5.

The results of linear regression analysis and prediction accuracy of concentrations of Ni, Zn, Cu, As and Pb by the PXRF method against chemical analysis were shown in Fig 5. The determination coefficients (R^2^) of Zn, Cu, Pb, As and Ni were 0.99, 0.95, 0.92, 0.73 and 0.37, indicating that Zn, Cu and Pb were well predicted by the PXRF method. PXRF could quantitatively predict the concentration of As in soil; the results for Ni still need substantial improvement, as only qualitative predictions of Ni content could be obtained. Soil types, water content and soil particle diameter might affect the ability of PXRF to predict soil Ni concentration.

The prediction results for As had the smallest RMSE (6.56; Fig 5). The RMSE values of Pb, Cu and Zn were 12.03, 12.37 and 28.92, respectively. Ni had the largest RMSE of 33.04. RPD was in the order of Zn (9.35), Cu (2.88), Pb (2.53), As (0.92) and Ni (0.36). This indicated that the PXRF method had a robust ability to predict Zn concentration in soil. The prediction results for As and Ni still need to be improved.

### 3.4 Rapid assessment and classification of heavy metal pollution grade

In this study, first, VNIR was used to measure pH of soil samples and soil samples were classified by pH into three classes (pH <6.5, 6.5–7.5 and >7.5) with reference to the national soil environment quality standard [22] and then, soil samples were divided into the calibration dataset (201 soil samples) and the validation (100 soil samples) dataset. Second, we assessed the composite pollution grade of the validation set according to the measured pH value and heavy metals concentration. Then PXRF was used to test the pH value and heavy metals concentration of soil samples in the validation set. After that the test results of PXRF and pH classification results were combined to assess the composite pollution grade and finally the assessment results of PXRF and laboratory measurement were compared and tested for consistency between the two methods; results of this comparison were shown in Table 5.

**Table 5:** Comparison of heavy metal pollution grade classification between PLSR and chemical analysis.

• **PP** ^a^	**Safety**	**Alert Limit**	**Slight Pollution**	**moderate Pollution**	**Severe Pollution**	**Total**	**Prediction Classification Accuracy(%)**
• **CT** ^b^							
**Safety**	**50**	4	0	0	0	54	92.59
**Alert Limit**	0	**20**	2	0	0	22	90.91
**Slight Pollution**	0	1	**21**	0	0	22	95.45
**Moderate Pollution**	0	0	0	**1**	1	2	50
**Severe Pollution**	0	0	0	0	**0**	0	100
**Total**	50	25	23	1	1	100	
**Method Classification Accuracy(%)**	100	80.00	91.30	100	-	**Kappa Coefficient:0.87**	

^a^ PP, PLSR Prediction;

^b^ CT, Chemical test.

According to the traditional chemical analysis results for 100 sample points in the validation set, the number of sample points whose comprehensive pollution level was safety, the alert limit, light pollution, moderate pollution and severe pollution were 54, 22, 22, 2 and 0, respectively (Table 5). Based on the test results of PXRF and VNIR, the PLSR model was used to classify the validation dataset into five grades according to NCPI. The prediction classification accuracies by PXRF and VNIR for soil composite pollution grade belonged to safety, the alert limit, slight pollution, moderate pollution and severe pollution were 92.59%, 90.91%, 95.45%, 50% and 100%, respectively, meanwhile the method classification accuracies of it were 100%, 80.00%, 91.30%, 100% and 0, respectively. The classification accuracies were generally at a very high level. The Kappa coefficient of the classification matrix was 0.87. This means that the evaluation of heavy metal pollution results by PXRF and VNIR spectroscopy was almost identical with the results of the traditional chemical analysis method (Table 5).

This result demonstrated that it is very reasonable and feasible to use PXRF and VNIR to rapidly evaluate soil heavy metal pollution grade. The main classification error that occurred was that some low pollution grade soil samples were wrongly classified to a higher pollution grade, which was mainly caused by the detection limits of the PXRF. These samples had heavy metal contents lower than the detection limits of the PXRF, so the values of the detection limits were used instead of assigning a 0 value, which obviously caused some overestimation of heavy metal content and increased the soil heavy metal pollution grades of these samples. In this study, limited by the detection limits of the PXRF, we analyzed concentrations of five heavy metals (Pb, As, Cu, Zn and Ni). This also had some effect on the results of soil heavy metals composite pollution assessment. With the development of a proximity sensor, the detection limits will be reduced and more and more heavy metal elements will be able to be detected by PXRF, indicating the great potential of this method.

## Conclusion

This study provides a method to verify the soil heavy metal pollution grades classification synthesizing PXRF and PVNIR. The main conclusions are as follows:

Restricted by the detection limits of the PXRF, only Ni, Cu, Zn, As and Pb could be analyzed via PXRF in practice. Rapid test results of soil heavy metals via PXRF could be influenced by water content, soil particle diameter, soil type and other factors [35]; these factors will be taken into consideration in further research.

The PLSR model with pretreatment by SG and SNV performed best in predicting pH while it showed a trend of slight underestimation. PXRF showed a robust prediction ability in Zn, Cu and Pb (RPD>2) while the prediction accuracy for Ni and As content still needs to be improved (RPD<1.4).

Based on the prediction results synthesizing PXRF and PVNIR, the PLSR model could feasibly be used to rapidly assess and classify heavy metal pollution grades. The Kappa coefficient of 0.87 showed that the prediction classification was highly similar to that of the traditional chemical analysis and classification result. With further development of PXRF spectrometers, the detection limits are likely to be lowered. Integrated use of PXRF and PVNIR to predict and assess soil heavy metals pollution is feasible and has broad potential application. It provides a theoretical foundation for rapid prediction and assessment of soil heavy metals pollution. Further study will be mostly focused on in situ evaluation of soil heavy metal pollution using PXRF and PVNIR to ensure the method is available and reliable in practice.

## Supporting information

S1 Table Laboratory measured value and PXRF measured value of soil heavy metals of soil samples. This table provides the measured results of heavy metals via laboratory analysis and PXRF method.
(XLSX)

S2 Table Reflectance of soil samples. This table contanins spectral reflectance of 301 soil samples (Themo XRF).
(XLSX)

S3 Table Comparison between the prediction accuracy of PLSR models with different spectral preprocessing methods. This table provides the prediction accuracies of five different spectral pretreatment methods(Savitzky-Golay denoising, SG; First-order difference, FD; Absorption rate, ABS; Multiple Scatter Correction, MSC; Standard Normal Variation, SNV).
(XLSX)
